# Loop-Mediated Isothermal Amplification (LAMP): A Rapid, Sensitive, Specific, and Cost-Effective Point-of-Care Test for Coronaviruses in the Context of COVID-19 Pandemic

**DOI:** 10.3390/biology9080182

**Published:** 2020-07-22

**Authors:** Robin Augustine, Anwarul Hasan, Suvarthi Das, Rashid Ahmed, Yasuyoshi Mori, Tsugunori Notomi, Bhavesh D. Kevadiya, Avnesh S. Thakor

**Affiliations:** 1 Department of Mechanical and Industrial Engineering, College of Engineering, Qatar University, Doha 2713, Qatar; rashid.ahmed@qu.edu.qa; 2 Biomedical Research Center (BRC), Qatar University, Doha PO Box 2713, Qatar; 3 Department of Medicine, Stanford University Medical Center, Palo Alto, CA 94304, USA; suvarthi.d@gmail.com; 4 Eiken Chemical Co., Ltd., Research and Development Division, Taito-ku 110-8408, Japan; yasuyoshi_mori@eiken.co.jp (Y.M.); tsugunori_notomi@eiken.co.jp (T.N.); 5 Interventional Regenerative Medicine and Imaging Laboratory, Department of Radiology, School of Medicine, Stanford University, Palo Alto, CA 94304, USA; kevadiya@stanford.edu (B.D.K.); asthakor@stanford.edu (A.S.T.)

**Keywords:** point-of-care tests, COVID-19, coronavirus, SARS-CoV-2, LAMP, RT-LAMP, reverse transcription loop-mediated isothermal amplification

## Abstract

The rampant spread of COVID-19 and the worldwide prevalence of infected cases demand a rapid, simple, and cost-effective Point of Care Test (PoCT) for the accurate diagnosis of this pandemic. The most common molecular tests approved by regulatory bodies across the world for COVID-19 diagnosis are based on Polymerase Chain Reaction (PCR). While PCR-based tests are highly sensitive, specific, and remarkably reliable, they have many limitations ranging from the requirement of sophisticated laboratories, need of skilled personnel, use of complex protocol, long wait times for results, and an overall high cost per test. These limitations have inspired researchers to search for alternative diagnostic methods that are fast, economical, and executable in low-resource laboratory settings. The discovery of Loop-mediated isothermal Amplification (LAMP) has provided a reliable substitute platform for the accurate detection of low copy number nucleic acids in the diagnosis of several viral diseases, including epidemics like Severe Acute Respiratory Syndrome (SARS) and Middle East Respiratory Syndrome (MERS). At present, a cocktail of LAMP assay reagents along with reverse transcriptase enzyme (Reverse Transcription LAMP, RT-LAMP) can be a robust solution for the rapid and cost-effective diagnosis for COVID-19, particularly in developing, and low-income countries. In summary, the development of RT-LAMP based diagnostic tools in a paper/strip format or the integration of this method into a microfluidic platform such as a Lab-on-a-chip may revolutionize the concept of PoCT for COVID-19 diagnosis. This review discusses the principle, technology and past research underpinning the success for using this method for diagnosing MERS and SARS, in addition to ongoing research, and the prominent prospect of RT-LAMP in the context of COVID-19 diagnosis.

## 1. Introduction

The emergence of a novel coronavirus disease (COVID-19) in late 2019 and its subsequent evolution into a pandemic created a severe global public health and economic concern. The pandemic has already affected more than 8.5 million people and resulted in the death of about 450,000 people within six months since the first reported case in Wuhan city, Peoples Republic of China [1,2]. The zoonotic infectious agent, known as Severe Acute Respiratory Syndrome (SARS) Coronavirus 2 (SARS-CoV-2), is reportedly transmitted among humans via respiratory droplets and aerosols generated from infected persons while sneezing, talking, and/or coughing [3]. Based on the first report on the clinical examination of patients, COVID-19 manifests with pneumonia and a set of symptoms which can include fever with or without a cough, fatigue, myalgia, sputum production, headache, hemoptysis, and/or diarrhea [2]. Complications consist of respiratory distress, severe cardiac injury, RNAaemia, secondary infection, and can even lead to death [2]. Thus, the pathological outcomes of COVID-19 were comparable with that of SARS and Middle East Respiratory Syndrome (MERS) infections [4]. However, as more reports come in, a major percentage of patients present to clinics with milder symptoms which can include olfactory dysfunction [5] or a multisystem mild inflammation [6] that can initially go undetected, but can become severe over time. In addition to patients being mildly symptomatic or often asymptomatic, the aggressive community transmissibility sets COVID-19 apart from earlier episodes of respiratory viral diseases, and contributes to its current pandemic proportions [7]. Lack of a cost-effective, reliable testing method makes the scenario worse and urges researchers to develop and deploy a rapid, sensitive, highly specific, and inexpensive Point of Care Test (PoCT)s in order to successfully identify the infected persons early, provide the necessary medical support, and take mitigative measures to control the spread of this disease [8].

Since, SARS-CoV-2 belongs to the same class as MERS and SARS viruses, and since their etiologies mostly match, the approaches optimized for disease diagnosis during those epidemics can be considered for COVID-19 diagnosis with modifications [9]. Along with X-Rays [10] and Computed tomography (CT) imaging techniques [11], tests based on gene amplification by Polymerase Chain Reaction (PCR) like quantitative reverse transcriptase PCR (qRT-PCR) and RT-PCR have been widely recognized as standard confirmatory tests for coronavirus detection [12]. Despite the universal recognition of PCR-based assays as a gold standard for molecular detection of viral diseases by government and medical councils, these methods require highly skilled personnel and sophisticated equipment, which makes them impractical in the setting of developing countries with limited resources. Moreover, PCR-based methods require time-consuming and complicated protocols that limit their diagnostic efficacy in an active pandemic situation with rapidly and exponentially increasing number of incidences, specifically in populous regions of the world. Taken together, this necessitates substitution with an equally reliable molecular detection method for diagnosing and controlling COVID-19.

**Figure 1:**
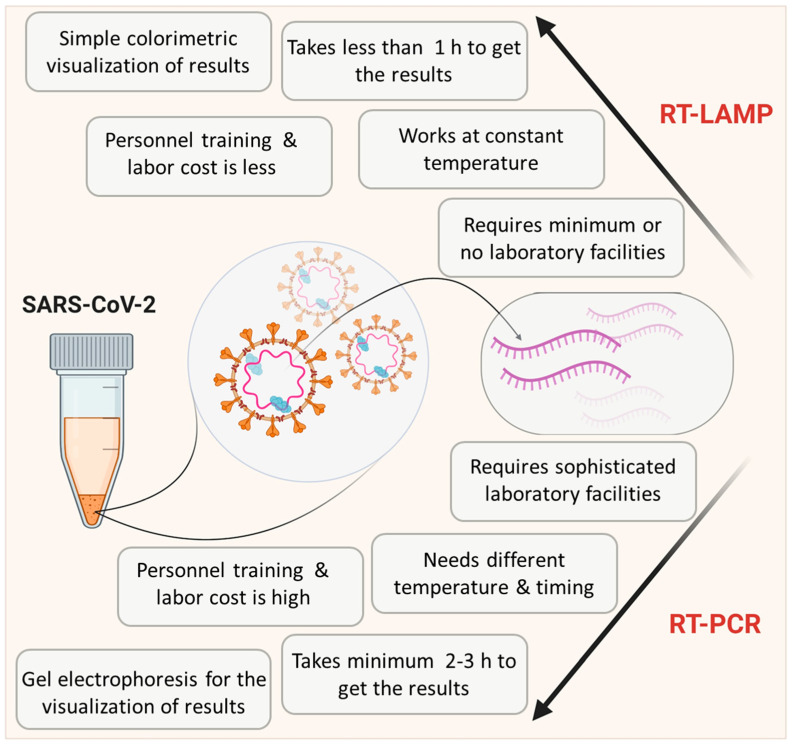
Scheme showing the comparison of reverse transcription loop-mediated isothermal amplification (RT-LAMP) with RT-PCR. Data is adapted from Dhama et al. [19].

One step loop-mediated isothermal amplification (LAMP) reaction is capable of detecting even a few copies of target nucleic acid sequences under isothermal conditions (usually 60–65 °C) with the help of specially designed primer sets. Due to its simplicity, it is gaining more popularity in the diagnosis of various viral diseases [13,14,15,16,17]. Detection of RNA viruses requires an additional reverse transcriptase enzyme and this method is then referred to as Reverse Transcriptase LAMP (RT-LAMP) [18]. Critical features of RT-LAMP in comparison with RT-PCR are depicted in Figure 1. Unlike PCR, this technique can be performed in a low-resource setting by merely heating the samples and reagents in a single reaction tube. As illustrated, RT-LAMP has clear advantages over RT-PCR as a PoCT in a pandemic scenario [19]. Indeed a simple visual inspection can even provide an idea about the outcome of the LAMP reaction without involving any specialized equipment [18,20]. The cost of LAMP per test is also considerably lower than other available molecular tests [21]. Thus, many researchers are focusing on the optimization and modifications of RT-LAMP methodology to match the requirements of COVID-19 diagnosis in laboratories with limited resources in developing countries, where uncontrolled and undetected spread of COVID-19 can result in an unpredictable outcome.

The first clinically approved LAMP reagent was used for SARS diagnosis in Japan [22], which indicates its potential as a PoCT for COVID-19. This review comprehensively discusses the reports on the diagnostic application of LAMP-based assays in past viral epidemics and the current COVID-19 pandemic. It also provides notes on the basic principle, procedures, and possible improvements in the existing LAMP-based assays and stresses on its advantages over other nucleic acid amplification-based methods. Furthermore, this article reveals the potential of using a LAMP assay as a simple and easy-to-use platform for the effective application as a PoCT tool for diagnosing COVID-19. 

## 2. Principle and Salient Features of the LAMP Method

**Figure 2:**
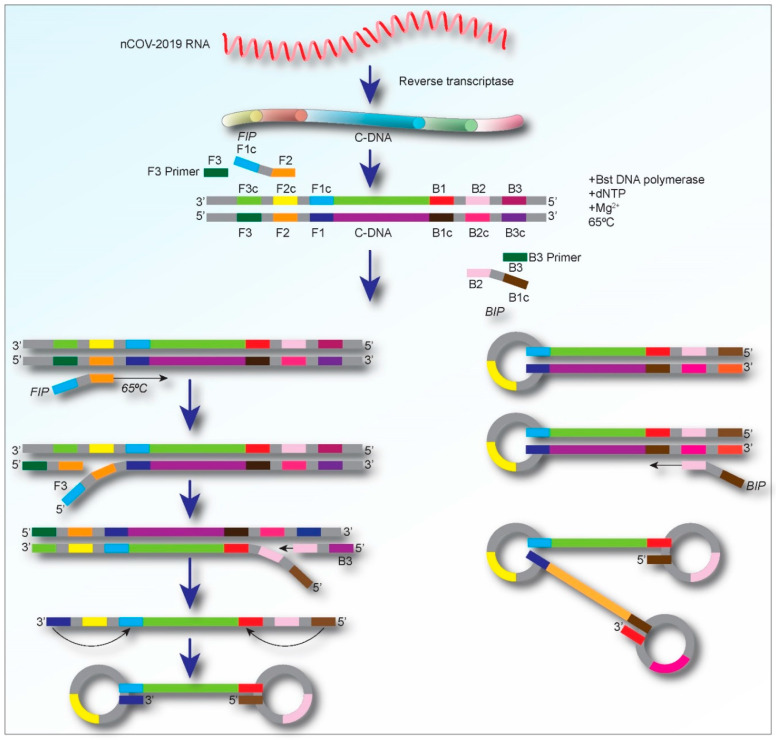
Schematic representation displaying the process of the amplification for the RT-LAMP assay. Initially, the LAMP primers bind to the complementary target cDNA sequences (in the case of RNA viral detection), and dumbbell-shaped DNAs are produced. Then, during the cycling amplification step, several copies of such dumbbell DNAs are continuously produced. The products formed during the cycling amplification step are used in the elongation phase to generate amplified DNA with various sizes.

The main components of LAMP reagents comprise of a set of salts, nucleotides, DNA polymerase, (which catalyzes the synthesis of complementary strands with strand displacement activity), and four to six primers including two loop primers (loop F and Loop B), forward and backward inner primers (FIP and BIP, respectively), and two outer primers (F3 and B3) [23,24]. For detecting RNA viruses, a heat-stable reverse transcriptase enzyme is also needed as an additional component [23]. A brief schematic representation of the details of the molecular-level process of the RT-LAMP assay is shown in Figure 2.

Development of a LAMP assay for the diagnosis of a specific disease starts with designing specific LAMP primers using online tools such as PrimerExplorer and LAVA (LAMP Assay Versatile Analysis) [25]. The primer sequences are represented as F3, F2, F1, B3, B2, and B1. Among these, F3 and B3 are the forward outer and reverse outer primers, respectively. The F2/B2 sequences and their complementary F1/B1 (F1c/B1c) sequences are referred to as FIP and BIP. Typically, four primer sequences are enough to amplify a target nucleic acid [26,27], but, to enhance the specificity and efficacy of the reaction, two additional loop primers (LoopF and LoopB) are incorporated in the LAMP reaction mixture [14]. The loop primers identify the area between the F2/B2 and F1/B1 in the sequence [27]. In addition to the primers, the LAMP methods require Bst polymerase, deoxynucleotide triphosphates (dNTPs), magnesium sulfate, betaine, and buffer solution for the enzyme [28]. The amplification products, which have a stem-loop DNA structure, contain numerous inverted repeats of the objective region and cauliflower-like structures with multiple loops (Figure 2). The LAMP assay can rapidly (within an hour) amplify and produce a large number of DNA copies, usually 100 times higher than conventional PCR, at 60–65 °C [29]. Moreover, the amplification of the target sequences, correlative to the initial quantity, can be assessed by a simple visual examination of the turbidity, which results from a white precipitation of magnesium pyrophosphate as a by-product of the reaction [20,30,31,32].

To obtain a more robust result, an inexpensive turbidimeter can be employed to quantify the turbidity at 650 nm in the reaction tube. The detection of LAMP amplicons can also be confirmed with several simple techniques such as agarose gel electrophoresis [33] by measuring fluorescence from the incorporated DNA intercalating agents such as SYBR Green I [34] or other modifications [35]. However, for the sake of rapidity, visual detection can be an immediate and straightforward option for laboratories without specialized equipment in the context of COVID-19 diagnosis [28]. A colorimetric visual inspection method has been developed where a suitable dye was dried in the caps of the reaction tubes to detect the amplified nucleic acids [36]. At the end of the reaction, amplicons were mixed with the dye and the color changes indicated the presence of a positive reaction.

## 3. LAMP for the Diagnosis of Viral Diseases

**Figure 3:**
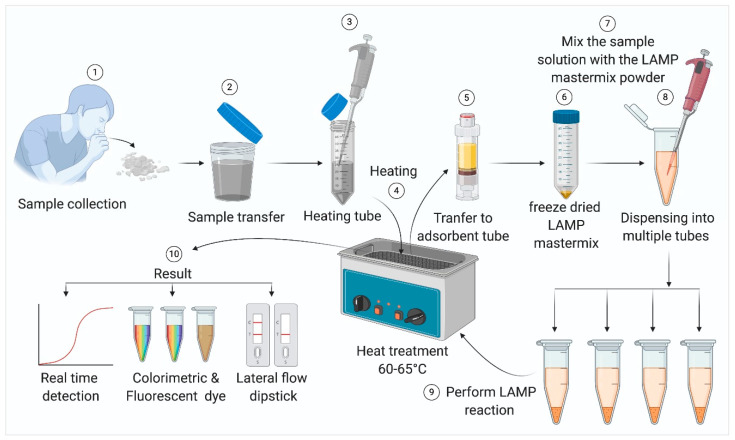
Steps in sample processing for the LAMP reaction, the LAMP reaction in a simple water bath, and the detection of amplicons.

LAMP diagnostic methods have been extensively used for detecting both DNA and RNA viral pathogens [37,38]. These assays have been developed and used for diagnosing several widespread human pathogenic agents such as human immunodeficiency virus (HIV) [39], Japanese Encephalitis virus [40], Chikungunya virus [16], human papillomavirus [41], Dengue virus [15,42], West Nile virus [43], and Mumps virus [44]. Highly pathogenic avian influenza (HPAI) H5N1 virus [45,46], SARS virus [47], and MERS virus [48]. As in the case of PCR tests, LAMP–based tests also start with the collection of samples from the patient. For respiratory viral diseases like SARS, MERS, and COVID-19, samples can be collected by a nasopharyngeal and/or oropharyngeal swab or sputum [49]. Notably, in case of patients where the infection is already lodged deeper in the respiratory tract, a sputum sample might present fewer chances of a false negative result. It also presents significantly lower risk of exposure for healthcare workers, as sputum can be self-collected by the patient under contactless supervision. A schematic representation of the standard operating procedure (SOP) for LAMP-based viral pathogen detection is provided in Figure 3. In case of contagious viral diseases, samples should be heat-inactivated before subsequent steps. Generally, adsorbent tubes are used to remove inhibitors present in the samples, which may interfere with the reaction. The samples or extracted viral nucleic acids are usually mixed with the LAMP reagents and heated at 60–65 °C for about 30 min to complete the amplification. 

In addition to detection of viral diseases [50], microbial infections have utilized LAMP-based detection, successfully, and often directly from clinical samples without isolating RNA [18,51]. While directly using patient samples, a single set of specific primers targeted toward the fusion-protein gene could produce amplicons in two hours. Cross-validation by conventional PCR strengthened the reliability of this system. Another remarkable advancement was the effective use of dry LAMP reagents that simplified the storage and handling of samples without compromising on the sensitivity or specificity [52]. Such modifications can majorly contribute toward the development of the RT-LAMP as a suitable PoCT for COVID-19. Portable units of the RT-LAMP, which can deliver accurate results within 15 min, eliminate the need for multiple equipment-based steps that can also have immense potential during a pandemic [53].

All reports, so far, point toward the potential revolutionary role and benefits of modified RT-LAMP over conventional PCR as PoCT in the current COVID-19 pandemic and the likes of it in the future.

## 4. RT-LAMP for the Diagnosis of SARS

SARS coronavirus (SARS-CoV) is a zoonotic virus that reportedly appeared for the first time in Guangdong province, China, in 2003, which was transmitted to humans from bats, and eventually spread to 26 countries by infecting about 8000 people in the same year [54].

Notomi et al. first reported an effective RT-LAMP-based amplification and detection method for the SARS virus, which could detect as low as 10 viral RNA copies/test within 45 min [55]. In two consecutive reports, the first one using patient serum samples [56], and the next one using nasopharyngeal aspirates [57] of clinically diagnosed SARS patients, Poon et al. endorsed the use of the RT-LAMP as an on-site detection method over conventional RT-PCR due to its simplicity and considerably lower cost. In a different study, using throat and nasal swabs, and combinations, from patients in the Vietnam SARS epidemic, the RT-LAMP assay reportedly showed a 100-fold higher sensitivity with a detection limit of 0.01 PFU (plaque-forming units) in less than an hour [47].

Several simultaneous investigations successfully attempted to improve the efficacy and sensitivity of SARS virus detection over a conventional nucleic acid amplification-based method and immunoassays by designing directed primer sets [58,59,60], and also by simplifying the reaction readout [59,60]. Changes in visual detections were brought about by employing calcein dye [59] or a multiplexed colorimetric reaction [60]. Remarkably, these improvements reflected upon the ‘sample-to-answer’ time being brought down to less than 30 min.

In light of these reports, RT-LAMP can be viewed as an accessible PoCT or near PoCT method for clinical testing in developing countries.

## 5. RT-LAMP for the Diagnosis of MERS

The MERS coronavirus (MERS-CoV), which is a beta coronavirus linked to a severe form of respiratory disease with a high fatality rate (~35%), was first reported in Saudi Arabia in 2012 [61] and spread beyond the Middle East [62]. Like most of the other known coronaviruses, MERS-CoV was also zoonotic in origin and was transmitted to humans from dromedary camels [63].

The first report using primers targeting nucleocapsid nucleotide sequences of MERS coronavirus implied that RT-LAMP should be chosen over RT-PCR for rapid diagnosis due to better sensitivity (0.7 copies of viral RNA detection compared to 1.6 copies) in less time (30 min) [64]. Later, this same group reported an improved visualization technique using quenching probes in place of the formerly used fluorescence-based detection [65]. A subsequent study corroborated the sensitivity (0.02–0.2 PFU detected in supernatants of infected cells) factor and validated the addition of one-step strand displacement probes (OSD) for real-time sequence-specific detection of LAMP amplicons. Further improvement in sensitivity and deployability was brought about by using one-pot RT-LAMP, and six targeted primers, which could detect as few as 0.4 copies of the virulent genome using a micro-chamber device [66].

A more straightforward and hassle-free method was established by combining the RT-LAMP assay with a vertical flow visualization strip (RT-LAMP-VF) [67]. After the amplification, the amplicons labeled with biotin and fluorescein isothiocyanate (FITC), which were present in two loop primers. The biotin-labeled amplicons could then bind to the colloidal gold particles, conjugated with streptavidin, and form a complex that was detected by the anti-FITC antibody (coated on the test line of the strip) as a visible colored line. 

Although there is no clear evidence for the widespread use of the RT-LAMP method as a PoCT in a clinical setting for the diagnosis of MERS, the results of the past studies suggest that the modified RT-LAMP assays can be used as efficient PoCT for the fast diagnosis of this highly infectious disease. 

## 6. RT-LAMP for the Diagnosis of COVID-19

**Figure 4:**
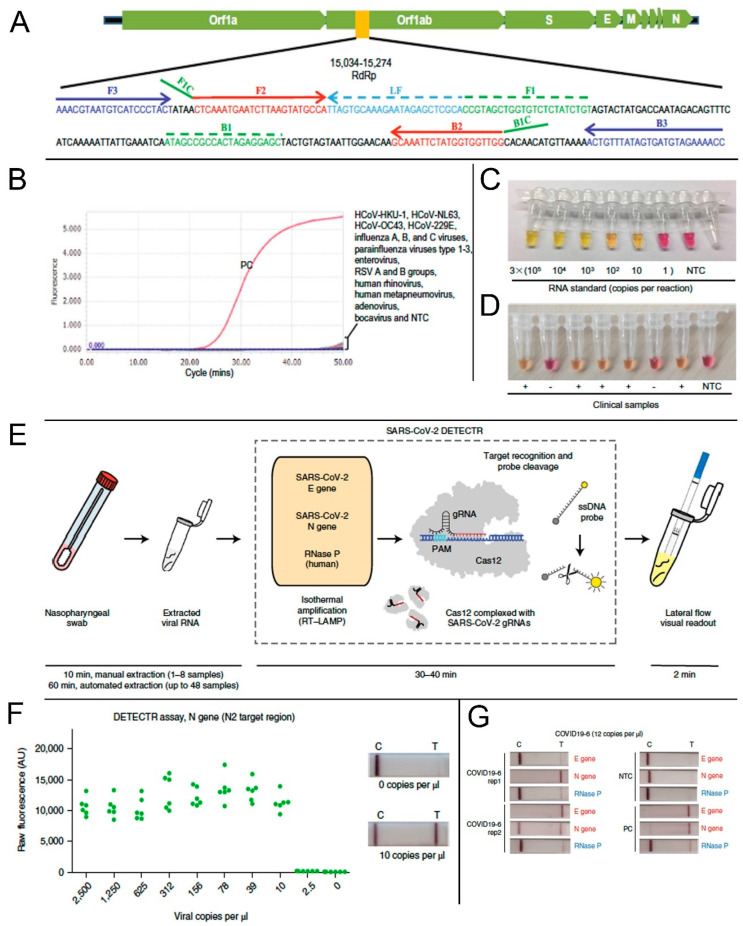
Application of reverse transcription loop-mediated isothermal amplification (RT-LAMP) method for the detection of SARS-CoV-2. (**A**) Location of RdRp primer sequence in the SARS-CoV-2 genome. (**B**) Cross-reactivity test of the novel SARS-CoV-2 RT-LAMP assay to other common respiratory viruses. Viral RNA isolated from a COVID-19 positive patient was used as a positive control (PC). NTC, non-template control. (**C**) RT-LAMP based colorimetric visual detection of SARS-CoV-2 RNA standards. (**D**) Clinical samples from positive (+) or negative (−) COVID-19 cases. NTC: non-template control. (**E**) Schematic representation of the SARS-CoV-2 DETECTR method workflow such as RNA extraction. The DETECTR method that involves the LAMP preamplification, and Cas12-based gene detection, and visualization by a fluorescent reader or lateral flow strip (TwistDx). (F) LoD for DETECTR assay showing the fluorescence values using SARS-CoV-2 DETECTR assay (*n* = 6) using SARS-CoV-2 N2 gene in vitro-transcribed (IVT) RNA. Representative lateral flow results for the assay of samples with 0 copies per μL and 10 copies per μL viral RNA. (**G**) Lateral flow strips showing SARS-CoV-2 DETECTR assay results after 3 min of flow. **A**–**D** are reproduced with permission from Lu et al. [70]. **E**–**G** are reproduced with permission from Broughton et al. [83].

According to the Center for Disease Control and Prevention (CDC) guidelines, swabs from the nasopharynx swabs, anterior nares, and mid-turbinate (latter two for symptomatic patients) can be used as samples for COVID-19 diagnosis in a healthcare setting. However, the idea of using the RT-LAMP reaction for the rapid detection of COVID-19 started arguably with a report from El-Tholoth et al. in early February 2020 [68]. Since this study was performed before COVID-19 assumed pandemic proportions, they could not use actual clinical samples. Instead, they used a synthetic DNA sequence of the viral agent, then called novel coronavirus (or nCoV), to test the feasibility of RT-LAMP application. Later, simulated patient samples viz. human saliva, serum, oropharyngeal swabs, nasopharyngeal swabs, and urine samples, spiked with COVID-19 nucleic acid sequences, were tested using the LAMP method [69]. Subsequent studies focused on the approaches to improve the sensitivity and specificity of the assay. One such study indicated that the primers designed for the RNA-dependent RNA polymerase sequence (RdRp sequence) of the Open Reading Frame 1ab (Orf1ab) polyprotein region of viral RNA showed higher amplification efficiency [70]. The RdRp primer information is included in Figure 4A. The assay showed high specificity against clinical specimens positive for several other known respiratory viruses (Figure 4B). Some other researchers reported the use of targeted primers for varied structural protein encoding regions like the spike-protein encoding S gene, or nucleocapsid encoding N gene, separately or in combination, with the common aim of achieving results with higher sensitivity, specificity, and minimal cross-reactivity in a short amount of time [71,72,73,74]. Primers targeting the Nsp3 gene in combination with those targeting N and S genes generated significantly satisfactory results and registered the shortest threshold time for cDNA production [73]. To further improve the efficacy of RT-LAMP reaction as a PoCT, researchers used several colorimetric detection methods where color-changing reagents were incorporated in the reaction mixture. A master mix composed of a pH-sensitive indicator dye (cresol red) was evaluated for the rapid RT-LAMP-based visual detection of COVID-19 [70,75,76]. This dye changes its color from red to orange-yellow as an indication of a positive reaction (Figure 4C,D) [77]. In positive samples, the pH of the LAMP reaction mixture tends to decrease because of higher DNA polymerase activity. Such a colorimetric RT-LAMP method utilizing phenol red (pH indicator), which changes color from pink to yellow at low pH, can also be used as a simple detection method in resource limited settings [78,79]. Moreover, rapid visual detection of a positive reaction can be done by using a LAMP master mix supplemented with SYTO^®^-9 (Thermo Fisher S34854, a double-stranded DNA or dsDNA binding agent), or leuco-crystal violet (that changes from colorless to violet on contact with dsDNA) [36,80].

Recent investigations focus on simplifying the procedure even further by incorporating everything in a ‘one-step’ or ‘single-tube’ assay using nanoparticle-based biosensors [81] or by including a magnetic bead capture step during processing of dry swabs to maximize viral RNA yield [81,82]. Although such advances are highly promising, further optimization studies are required to improve sensitivity and specificity. Numerous and various clinical samples need to be tested as well to satisfactorily demonstrate the consistent clinical applicability of these modified approaches. 

Most recently, Broughton et al. successfully reconfigured a DNA Endonuclease Targeted CRISPR Trans Reporter (DETECTR) platform using a visual lateral flow strip format to rapidly and accurately detect SARS-CoV-2 [83]. A schematic representation of the mechanism is provided in Figure 4E. This device used isothermal preamplification of primers based on protocols validated by government regulatory bodies. The system was able to provide the results within 30 min (from sample collection to result) with high sensitivity (10 copies of RNA per μL) (Figure 4F). To ensure the maximum specificity of detection, they used a high-fidelity CRISPR detection enzyme and designed sets of Cas12 guide RNAs (gRNAs) that can either differentiate SARS-CoV-2 or provide multi-coronavirus strain detection. The lateral flow strip format was highly effective to detect even a very low concentration of viral RNA per μL (Figure 4G).

In an effort to develop inexpensive apparatuses (that can be manufactured at large scale), an interesting study reported the development of a 3D-printed incubation chamber for performing RT-LAMP reactions and for successfully detecting SARS-CoV-2 in commercially available Eppendorf PCR tubes [84]. Conceivably, along with essential research efforts from various academic and research institutions across the world, public-private partnerships for bulk production of necessary equipment and reagents can help in implementing RT-LAMP as a PoCT for the rapid diagnosis and mitigation of the COVID-19 pandemic.

## 7. Challenges and Prospects

**Figure 5:**
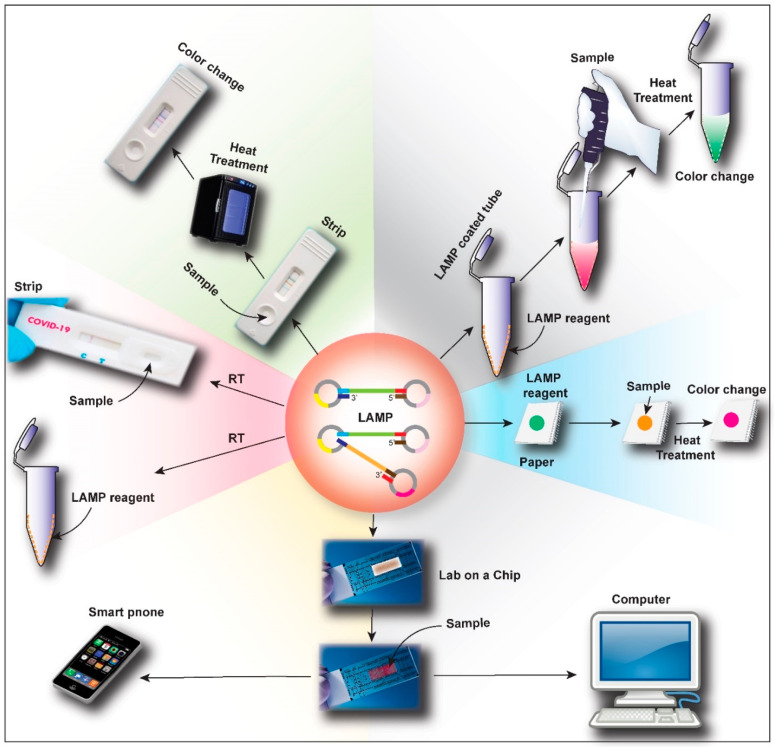
Schematic representation of the future possible advances in RT-LAMP based point-of-care test (PoCT) for novel coronavirus disease (COVID-19). LAMP reagents can be loaded in a paper-based system for easy use, which can be heated after sample loading to get quick results. Another advancement will be the coating of the LAMP reagent on the walls of a reaction tube where a simple mixing of the sample with the buffer and heating will give the results. Similarly, an RT-LAMP test-strip, like a conventional pregnancy detection kit, can also be developed. The researchers will also explore the possibility of developing an RT-LAMP master mix that can react at room temperature, which will fully transform the current concept of RT-LAMP based diagnosis. Most advanced development will be a lab on a chip (or LAMP on a chip) with advanced microfluidics, sensors, and computer integration, which can also be connected with an electronic device like computer or mobile phone to analyze the results.

Recent advances in LAMP technology suggest that there has been considerable progress in detecting target sequences with high specificity and sensitivity, which will help in the rapid diagnosis of a large population of suspected patients in a pandemic like COVID-19. Although the method is well-established with numerous recent modifications to match the disease-specific and pandemic-specific requirements, some challenges remain. The main disadvantage of LAMP-based method is the complexity of primer design [85] to achieve the specificity of a PCR test while providing the advantage of performing the whole experiment at a fixed temperature. Another possible limitation of such methods is that it can generate false-positive results due to the carry-over from previous experiments (due to its high sensitivity), especially when upgraded into an automated platform [86]. Notably, RT-LAMP detection by quenched fluorescent primers can overcome such limitations [87]. Several further modifications of the LAMP method to match the requirements of the COVID-19 pandemic are at the optimization and validation stages in various laboratories across the world. A schematic representation of such possible modifications is provided in Figure 5.

Although there is a challenge of keeping individual components in a non-reactive state, one of the prospective advancements is using reaction tubes with dry master-mix coated on the inner walls or caps. This can save time, which allows the lab technicians to omit adding different reagents individually to the reaction tube. Use of dry reagents also overcomes the requirement of cold storage facilities and temperature-controlled shipping [88,89]. In order to achieve fast result readouts, the LAMP technique can be multiplexed with polymeric beads tagged with specific optical signatures for barcoding [90]. Barcoding is already used for research but implementing the same for COVID-19 diagnostic purposes requires proper validation and scaling up. For easy digitalization of results and data management, computer integration of optical sensors for barcode scanning or use of nanomaterials that change electrical or magnetic properties upon binding to target sequences can prove beneficial [91,92,93]. Another report mentions analyzing the color change of gold nanoparticles in a salt solution on amplification of the viral genomic DNA [94].

A paper-based LAMP system for COVID-19 diagnosis has already been proposed by Yang et al. [95]. With some modifications, this can be transformed into a diagnostic tool [74,96,97] for use by any home-quarantined person or an individual with limited access to healthcare facilities, who can perform a self-test and share the results with healthcare professionals for remote analysis. Moreover, a strip-based rapid and accurate test, like the reconfigured DETECTR platform from Mammoth Biosciences, in combination with a glucometer-like readout machine, can completely revolutionize the concept of pandemic monitoring with home-based diagnosis [83]. A caveat of widely deploying LAMP-based diagnostic tools in home settings, however, is to ensure workability of the entire reaction at room temperature, which is difficult, but achievable.

A recent study demonstrated the possibility to fully automate LAMP methods for the rapid and effortless molecular diagnosis [98], which may pave a way to the automation of RT-LAMP based COVID-19 diagnosis. In this system called “Simprova” (Developed by Eiken Chemical Co., Ltd.), a central unit that controls the entire system, a pretreatment unit that isolates and purifies nucleic acid from samples, and the LAMP component to detect and amplify nucleic acid sequences were all integrated. Such systems (with inclusion of RT-enzyme) or microfluidic devices [90,99,100,101] can be cost-effective, portable, and can prove extremely effective in generating fast results using minimal samples and reagents. This is more likely when connected to an electronic device like computer or smartphone for real-time transmission to a pandemic monitoring center [102].

Thus, despite the existing shortcomings, RT-LAMP based methods have tremendous potential as a PoCT to meet the current diagnostic challenges of COVID-19.

## 8. Conclusions

As already reported for the effective diagnosis of several viruses, including coronaviruses causing SARS and MERS, RT-LAMP has been widely investigated for its vast potential in diagnosing and controlling the COVID-19 pandemic in developing countries. In addition to serving as a life-saving tool for large populations residing in parts of the world without access to modern diagnostic facilities, it would not be wrong to suggest that even developed countries struggling to control the rampant spread of the disease can benefit immensely from implementing modified RT-LAMP-based PoCT or home-based diagnostic tools. This method understandably has significant advantages over conventional RT-PCR in a pandemic scenario like the present one.

Ideally, for easy and largescale deployment, the detection modules must be miniaturized and integrated with the on-chip RT-LAMP system. In conclusion, the integration of the RT-LAMP method with optical and nanomaterial-based system and/or advanced information technologies can help realize a PoCT for the rapid, sensitive, specific, and cost-effective diagnosis of COVID-19 by minimally trained individuals and with limited technical infrastructure in developing and developed countries alike.
